# Gene Circuit Analysis of the Terminal Gap Gene *huckebein*

**DOI:** 10.1371/journal.pcbi.1000548

**Published:** 2009-10-30

**Authors:** Maksat Ashyraliyev, Ken Siggens, Hilde Janssens, Joke Blom, Michael Akam, Johannes Jaeger

**Affiliations:** 1 Center for Mathematics and Computer Science, Centrum Wiskunde and Informatica, Amsterdam, The Netherlands; 2 Laboratory for Development and Evolution, University Museum of Zoology, Department of Zoology, University of Cambridge, Cambridge, United Kingdom; 3 EMBL/CRG Research Unit in Systems Biology, CRG–Centre de Regulació Genòmica, Universitat Pompeu Fabra, Barcelona, Spain; Université de la Méditerranée & INSERM U928 - TAGC, France

## Abstract

The early embryo of *Drosophila melanogaster* provides a powerful model system to study the role of genes in pattern formation. The gap gene network constitutes the first zygotic regulatory tier in the hierarchy of the segmentation genes involved in specifying the position of body segments. Here, we use an integrative, systems-level approach to investigate the regulatory effect of the terminal gap gene *huckebein (hkb)* on gap gene expression. We present quantitative expression data for the Hkb protein, which enable us to include *hkb* in gap gene circuit models. Gap gene circuits are mathematical models of gene networks used as computational tools to extract regulatory information from spatial expression data. This is achieved by fitting the model to gap gene expression patterns, in order to obtain estimates for regulatory parameters which predict a specific network topology. We show how considering variability in the data combined with analysis of parameter determinability significantly improves the biological relevance and consistency of the approach. Our models are in agreement with earlier results, which they extend in two important respects: First, we show that Hkb is involved in the regulation of the posterior *hunchback (hb)* domain, but does not have any other essential function. Specifically, Hkb is required for the anterior shift in the posterior border of this domain, which is now reproduced correctly in our models. Second, gap gene circuits presented here are able to reproduce mutants of terminal gap genes, while previously published models were unable to reproduce any null mutants correctly. As a consequence, our models now capture the expression dynamics of all posterior gap genes and some variational properties of the system correctly. This is an important step towards a better, quantitative understanding of the developmental and evolutionary dynamics of the gap gene network.

## Author Summary

Currently, there are two very different approaches to the study of pattern formation: Traditional developmental genetics investigates the role of particular factors in great mechanistic detail, while newly developed systems-biology methods study many factors in parallel but usually remain rather general in their conclusions. Here, we attempt to bridge the gap between the two by studying the expression pattern and function of a particular developmental gene—the terminal gap gene *huckebein (hkb)* in the fruit fly *Drosophila melanogaster*—in great quantitative detail using a systems-level approach called the gene circuit method. Gene circuits are mathematical models which allow us to reconstitute a developmental process in the computer. This allows us to study the function of the *hkb* gene in its wild-type regulatory context with unprecedented accuracy and resolution. Our results confirm earlier, qualitative evidence, and show that *hkb* plays a small, but crucial role in gap gene regulation. Understanding *hkb*'s regulatory contributions is essential for our wider understanding of dynamic shifts in the position of gap gene expression domains which play important roles during both development and evolution.

## Introduction

How genes contribute to pattern formation is one of the central questions of modern developmental biology. Traditionally, this question has been addressed using genetic and molecular approaches. Although very powerful, these approaches have several important limitations: First, it is difficult to study expression features which are not specifically affected by a particular mutation (see below). Second, there is always some remaining ambiguity whether an interaction is direct or not [1]. And finally, it is difficult to establish whether known regulatory interactions are not only necessary, but also sufficient to account for patterning in the wild-type system [2]. It is important to develop complementary approaches that help us to overcome these limitations. Here, we show how such an approach can be used to investigate the patterning function of a particular gene in its wild-type context in a rigorous and quantitative manner.

The patterning system we study is the gap gene network of *Drosophila*. Gap genes constitute the first zygotic step in a regulatory cascade which leads to the determination of body segments along the major (anterior-posterior, A–P) body axis during the blastoderm stage, shortly before the onset of gastrulation [3],[4]. They are involved in the regulation of pair-rule and segment-polarity genes. The latter establish a segmental pre-pattern of gene expression by gastrulation time. Gap genes such as *hunchback (hb)*, *Krüppel (Kr)*, *giant (gt)* and *knirps (kni)* are expressed in broad, overlapping domains. These domains are established by spatial gradients of the maternal co-ordinate proteins Bicoid (Bcd), Hb, and Caudal (Cad) (reviewed in [5]). Later these expression patterns are maintained and refined through gap-gap cross-regulation (see [1], and references therein), as well as regulation by the terminal maternal system acting through the terminal gap genes *tailless (tll)* and *huckebein (hkb)* (reviewed in [6]). In this report, we focus on *hkb* and its role in gap gene regulation.

The expression domains of gap genes in the posterior region of the embryo shift towards the anterior over time [7]. These shifts are independent of maternal factors or gap protein diffusion. Instead, they are caused by an asymmetric cascade of cross-repressive interactions between gap genes with overlapping expression domains (reviewed in [8]): the posterior *hb* domain is established during late blastoderm stage; this leads to the repression of Hb's anterior neighbour *gt*; Gt then represses its anterior neighbour *kni*, whose protein product in turn represses its anterior neighbour *Kr*. In contrast, anterior neighbours never repress their posterior neighbours. Note that a qualitatively similar, but less specific, mechanism for domain shifts has been predicted previously based on theoretical considerations [9].

This mechanism suggests that the posterior *hb* domain plays a central role in the initiation and regulation of gap domain shifts. However, our understanding of *hb* regulation in this domain is poor and incomplete. In particular, the position of its posterior boundary itself shifts over time[10], but the regulatory mechanism by which this is achieved remains unknown. In this paper, we use the gene circuit method—a data-driven modelling approach [11],[12]—to investigate the role of Hkb in the establishment and subsequent shift of the posterior *hb* domain.

**Figure 1:**
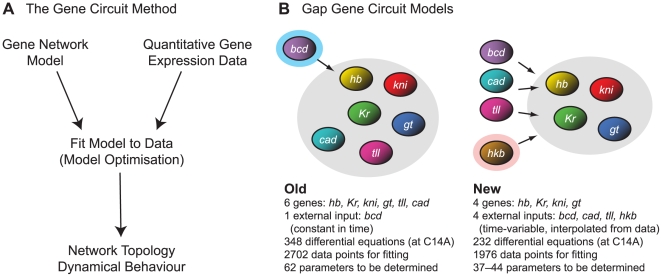
The gene circuit method: old vs. new models. (A) Reverse engineering gene regulatory networks using the gene circuit method: A mathematical (dynamical) model of the network is fit to quantitative, spatial gene expression data using combined global and local non-linear optimisation approaches. The resulting gene circuits, consisting of specific estimated sets of parameter values, define regulatory interactions among genes within the network (its regulatory topology). This topology is not defined a priori, but is extracted from the quantitative expression data by the fitting procedure. The resulting dynamical behaviour of the system can be analysed using graphical or numerical methods. (B) Previous gap gene circuit models used concentrations of the protein products of gap genes *hb*, *Kr*, *gt*, *kni*, *tll*, and of the maternal co-ordinate gene *cad* as state variables (grey shaded background), while the maternal protein gradient encoded by *bcd* was implemented as an external input which did not vary over time (6-gene models, left; time-constant Bcd indicated by blue shaded background). Current gap gene circuit models only include the trunk gap genes *hb*, *Kr*, *gt* and *kni* as state variables (grey shaded background), implementing *bcd*, *cad*, *tll* and *hkb* as time-variable external inputs since they are not regulated by gap genes themselves (4-gene models, right). *hkb* (highlighted), which is the focus of this study, has not been considered in previous models. See main text for details.

The gene circuit method uses mathematical models of gene networks as computational tools to extract regulatory information from quantitative, spatial gene expression data (Figure 1A). We obtained such data for *hkb* expression using a slightly modified version of an established data-processing pipeline (see [13], and references therein): (1) A polyclonal antibody against Hkb protein was raised and used to stain blastoderm stage *Drosophila* embryos. (2) Embryo images were acquired using a confocal laser scanning microscope. (3) Image segmentation was applied to obtain numerical tables of average protein concentrations per nucleus. (4) Embryos were sorted into time classes—each covering about 7 min of developmental time—based on Eve expression and morphological markers. (5) Non-specific background staining was removed and (6) data were averaged across all embryos stained for Hkb at a given time point. This yielded an integrated, quantitative time-series of Hkb expression patterns, which we combined with equivalent data for other gap genes from the FlyEx data base [14],[15] for modelling and model fitting (see below).

To simulate the dynamics of gap gene expression, we use gene circuit models (see Methods for equations) [11],[12]. Such models have been successfully used in the past to investigate gap gene expression and regulation [1], [7], [16]–[22]. Gap gene circuits consist of a row of dividing nuclei along the A–P axis of the embryo. Between nuclear divisions, gap proteins are synthesised and decay within each nucleus. In addition, gap proteins diffuse between neighbouring nuclei which are not yet separated by cell membranes at this stage [23]. The model incorporates a few basic assumptions about eukaryotic transcriptional regulation: Regulatory input is fed into a sigmoid regulation-expression function. We assume that each regulatory interaction can be either repressive (if it is negative), absent (if it is close to zero) or activating (if positive), and hence can be represented by a single number or parameter in the model. For simplicity, we assume that regulatory inputs are additive and independent of regulatory context (i. e. the presence or absence of other regulators).

Previous gene circuit models included the gap genes *hb*, *Kr*, *kni*, *gt* and *tll* as well as the maternal co-ordinate gene *cad* (6-gene models; Figure 1B, left) [1], [7], [17]–[20]. All of these genes regulate and are regulated by other genes in the model. However, it is known from the experimental literature that neither *tll* nor the maternal contribution to *cad* are affected by gap genes (zygotic *cad* expression is repressed by Hb, but does not play a role in gap gene regulation) [24]–[29]. This can create modelling artifacts—inconsistent with experimental data—such as an expansion of *tll* expression which influences gap gene expression in the central region of the embryo [1],[21],[22]. It also leads to problems with the determinability of parameters involved in *tll* and *cad* regulation, which in turn affects determinability of regulatory parameters for other gap genes (see below) [19]. Finally, the absence of Hkb in these models results in incorrect expression and regulation of the posterior *hb* domain [1].

To avoid such problems, we use a revised model—first introduced in [21],[22]—which represents *tll* and *cad* as time-variable external inputs. This model only considers *hb*, *Kr*, *kni* and *gt* as core regulators of the network (4-gene models). Protein concentrations of the products of these genes constitute the state variables of the system, while levels of Tll and Cad are now calculated from data. It is assumed that they regulate, but are not themselves regulated by gap genes. These published models have a constant Bcd gradient and did not consider Cad data from late time points just before the onset of gastrulation [21],[22]. In contrast, we implement Bcd as a time-variable input, and use late Cad expression data to represent the rapidly changing expression dynamics of these two genes at that stage. Bcd starts being rapidly degraded right before the onset of gastrulation [10]. At the same time, Cad disappears from the central region of the embryo and refines into a posterior stripe of zygotic expression which has a homeotic, rather than maternal co-ordinate function [30]. Finally, the most important addition to the model in the context of this paper is that of the terminal gap gene *hkb*. Similar to *tll*, it is not regulated by gap genes itself [26],[28] and is included as yet another external input factor. Core regulatory genes and external inputs in our current 4-gene models are summarised in Figure 1B (right).

The modelling framework outlined above does not predetermine any specific regulatory interactions within the gene network. Instead, these interactions—and hence the regulatory topology of the network—are obtained by fitting the model to the data (Figure 1A). This is achieved by minimising a cost function that measures the difference between the two. Previous studies using gap gene circuits used a cost function based on the sum of squared differences between gap protein levels in the model and the data (ordinary least squares, OLS) [1], [7], [17]–[22]. However, the OLS cost function is an appropriate measure under certain assumptions only: all errors in the data have to be independent of each other, and are assumed to follow a normal distribution with zero mean and constant standard deviation. The latter condition clearly does not hold for our data set, since standard deviations vary for each gene over space and time (Figure S1) [10],[31]. Generally, standard deviations become smaller at late time points. They are also relatively small around domain boundaries, and almost negligible in non-expressing regions, indicating that domain position is determined with little variation towards the onset of gastrulation [10]. Therefore, it is more appropriate to consider data variability for model fitting by using a weighted least squares (WLS) cost function for optimisation (Maximum Likelihood Estimation, [32]), in which each squared difference between model and data is weighted inversely proportional to the standard deviation of the corresponding data point. In other words, data points with little embryo-to-embryo variability contribute more to the measured difference between model and data than those with a high variability between embryos. Here, we compare results obtained by both OLS and WLS fits to demonstrate that indeed, WLS is a more suitable measure than OLS not only in theory, but also in practice.

The resulting models are analysed in various ways to gain new biological insights. Analysis of the dynamical behaviour of our models allows us to associate specific regulatory interactions and mechanisms with specific features of gene expression (such as the establishment of a new expression domain or the formation, sharpening or shift of an expression domain boundary). This can either be achieved by graphical examination of specific interactions in the model [1],[2],[7], or by characterising the convergence of the system towards its various dynamical attractors [21],[22]. In addition, we can test how reliably our models predict a specific regulatory network topology, by statistical determinability analysis of our parameter estimates. This is achieved by calculating confidence intervals around our estimated solutions, which give us a range of values in which the true solution of our optimisation problem lies with a given probability (see Methods and [19],[33], for details). If these intervals do not range across several regulatory categories (‘activation’, ‘repression’, or ‘no interaction’), the parameter is well-determined. In contrast, if they cover more than one regulatory category, the parameter is only weakly determined, or not determined at all. It has been shown that biological network models always contain at least a few parameters which cannot be determined, and that this is usually due to parameter correlations [34]. Here, as in a previous study [19], we analyse such parameter dependencies by calculating an average correlation matrix across solutions.

In the sections that follow, we analyse the protein expression pattern of *hkb* in a quantitative manner. We then use these quantitative expression data as external input to new gap gene circuit models. We obtain parameter estimates for these models (and hence a predicted regulatory topology for the gap gene system) using fits with both OLS and WLS cost functions. We show that the latter produces more consistent and well-determined parameter estimates. In contrast to earlier models, these circuits now reproduce expression dynamics in the posterior *hb* domain correctly. In particular, they show a correct anterior shift in this expression domain, and thus correct shifts in all gap domains in the posterior region of the embryo. We analyse the dynamical behaviour of our model to show that this is due to the repressive influence of Hkb on *hb*. We further establish that this is the only significant contribution *hkb* makes to pattern formation by gap genes. The role of *hkb* as revealed by our models is entirely consistent with evidence from the experimental literature. Finally, we discuss its implications for gap domain shifts, segment determination and the evolution of the gap gene system.

## Methods

### Hkb Antibody

Polyclonal antiserum against Hkb protein was raised as follows: A full-length cDNA clone of *hkb* (FlyBase ID: FBgn0001204) was obtained from the *Drosophila* gene collection (http://www.fruitfly.org/DGC), and recombined into a pET-DEST42 GATEWAY expression vector (Invitrogen). The resulting construct was auto-induced in *E. coli* strain BL21(DE3) using Overnight Express medium (Novagen/Merck). 6xHis-tagged Hkb protein was purified according to [35]: The most prominent protein band was excised from a preparative SDS-PAGE gel and recovered by electroelution followed by dialysis against double distilled water. Antibodies were raised in two rats using 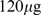 of protein per rat (Eurogentec).

### Quantitative Expression Data

Blastoderm stage embryos of *Drosophila melanogaster* (collected 1–4 hrs after egg laying) were stained against Hkb (dilution: 1∶100), Eve (1∶2000) and either Hb (1∶1000) or Kni (1∶400), using antisera described above (for 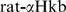), in [36] (for 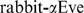) and in [35] (for 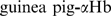, and 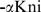). Eve is used for time classification [13]. As secondary antibodies, we used 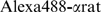, 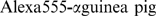 and 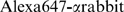 (Molecular Probes) at a dilution of 1∶4000. Nuclei were counter-stained using Hoechst 34580 (Invitrogen). Laterally oriented embryos were scanned using a 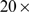 water-immersion objective on a Leica SP5 confocal scanning laser microscope. Fluorescent dyes were excited with a single wavelength at a time to prevent bleed-through between channels. The following wavelength windows were used for detection: 410–485 nm (with the 405 nm blue diode laser line), 495–555 nm (488 nm Argon), 565–625 nm (561 nm DPSS), and 640–720 nm (633 nm HeNe). To ensure reproducibility of measurements, scans were performed using identical detector gain and offset for all embryos on a slide. Images of dorsal nuclear and membrane morphology for time classification were obtained using differential interference contrast (DIC) with a 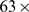 water-immersion objective.

**Figure 2:**
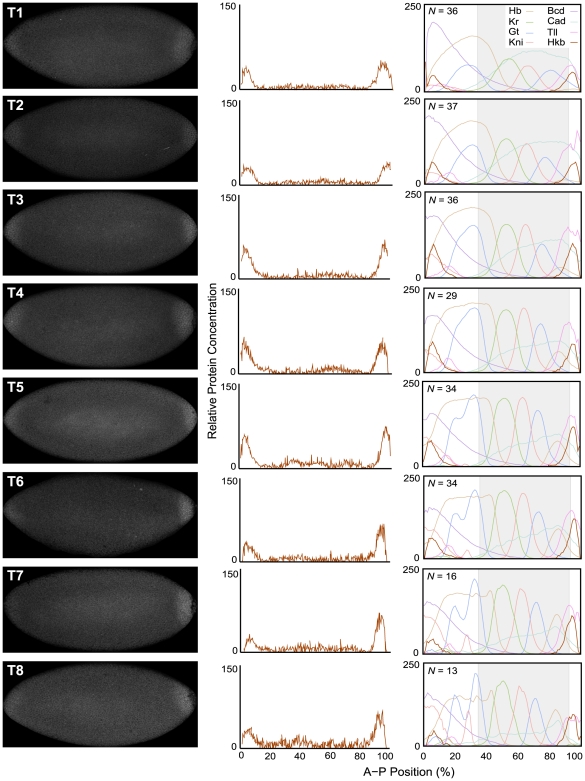
Quantitative analysis of *hkb* expression. This figure shows images of representative embryos stained against Hkb protein for each time class (T1–T8) during cleavage cycle 14A (left), with their corresponding quantified Hkb expression profiles (middle). Integrated Hkb expression data for each time class are shown, and compared to integrated profiles of Bcd, Cad, Hb, Kr, Gt, Kni, and Tll from the FlyEx data base [14],[15], on the right. *N* indicates the number of embryos on which each integrated Hkb pattern is based. Horizontal plot axes represent percent A–P position (where 0% is the anterior pole). Grey shaded background (on the right) indicates the trunk region of the embryo, which is covered by gap gene circuit models. Vertical plot axes show relative protein concentration (based on fluorescence intensity on an 8-bit range of 0 to 255). Integrated Hkb patterns have been scaled to facilitate comparison to other expression profiles. See Methods for details on time classes and data quantification.

Embryo images were processed to yield integrated expression data as described in the Introduction and in [13] (and references therein), with the following exceptions: (1) Images of embryos at early blastoderm stage (comprising cleavage cycles 9 to 13 (C9–C13); cleavage cycle 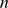 is the period between mitoses 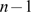 and 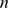 [23]) were segmented using a threshold-based algorithm: Images were de-speckled using a median filter; a top-hat transformation was used to remove uneven background; automated thresholding (using Otsu's method) was corrected interactively wherever necessary until all nuclei in an image were captured by the algorithm; finally, a watershed segmentation algorithm was applied to the distance transform of the thresholded image to avoid fused nuclei [37]. (2) Images of embryos at late blastoderm stage (cleavage cycle 14A (C14A)) were segmented using a watershed algorithm combined with nuclear edge detection as described in [38]. To reduce over-segmentation, we introduced an extended-minima transform before the watershed algorithm was applied [37]. (3) Expression data were not registered, as registration based on expression features in the central region fails at the termini where *hkb* is expressed, and not enough replacement features were available in that region of the embryo. (4) Due to its low signal-to-noise ratio, Hkb serum had to be used at a relatively high concentration (see above) to elicit a clearly detectable signal. This created high levels of non-specific background staining in the central region of the embryo, which our background removal procedure failed to completely remove. The residual central signal is clearly separated from the two expression domains at the termini. It does not seem to represent any real expression, and has not been observed in any previous study of *hkb* [26],[28],[39],[40]. To avoid modelling artifacts like those described for Tll in the Introduction, this signal was removed from integrated data by setting Hkb levels in the central region to zero. Moreover, integrated Hkb data were scaled (by an arbitrary factor of 3 across all time classes) to facilitate visual comparison (in Figure 2, right column) and to reduce numerical stability problems when solving the model (see below). Hkb expression data will be integrated into the FlyEx database, available at http://urchin.spbcas.ru/flyex or http://flyex.ams.sunysb.edu/flyex [14],[15].

Quantitative integrated expression data for Bcd, Cad, Hb, Kr, Kni, Gt and Tll are taken from the FlyEx database. Concentration measurements were taken at C13, as well as eight regularly spaced time points during C14A (T1–T8) [13]. The data set used for model fitting consists of 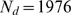 averaged nuclear protein concentrations. Averaging is achieved by collecting measurements from individual embryos into a number of bins along the A–P axis. Each integrated expression pattern at a given time point is based on data from 9–62 individual embryos (with the exception of Kni at C13, which is represented by 4 embryos only). Each embryo contributes measurements from multiple nuclei to a bin to be averaged. Therefore, the number of measurements used in the computation of the averaged concentration value per nucleus (the sample mean) is usually much larger than the number of embryos per time point. Based on this and the Central Limit Theorem [41], we assume that concentration values in averaged bins are approximately normally distributed. As it is not known how measurements are correlated, we take them to be independent of each other. Figure S1 shows integrated gap gene expression data with their associated standard deviations.

### Gene Circuit Models

Gene circuits are hybrid dynamical models with two continuous and one discrete rule: (1) interphase, (2) mitosis and (3) division [11]. During interphase, the change in concentration 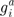 for each gap gene product 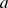 in each nucleus 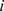 over time 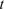 is described by the following system of ordinary differential equations (ODEs):
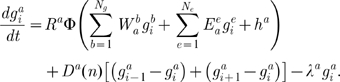
(1)The three terms on the right-hand side of the equation represent regulated protein synthesis, protein diffusion and protein decay. Integer indices 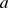 and 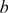 refer to regulated gap genes and regulators respectively, and 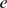 refers to external regulators. 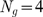 is the number of gap genes in the model (*hb*, *Kr*, *kni* and *gt*), 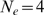 is the number of external regulatory inputs (provided by *bcd*, *cad*, *tll* and *hkb*, genes which regulate gap genes but are not regulated by gap genes themselves). 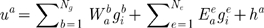 represents the total regulatory input to gene 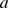. 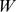 and 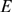 are genetic interconnectivity matrices (for state variables and external inputs respectively, of size 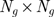 and 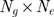) whose elements (called regulatory weights) each define one particular regulatory interaction in the gap gene network. 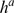 is a threshold parameter (which represents the influence of uniform maternal factors on the expression of gene 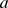) for the sigmoid regulation-expression function
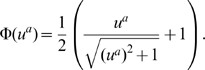
(2)Negative regulatory input 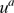 leads to increasing repression (with leakage), while positive regulatory input leads to increasing activation until saturation of gene expression at maximum production rate 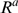. 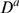 is a diffusion rate that depends on the distance between nuclei, which halves at every nuclear division (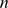 is the number of previous divisions). 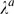 is the rate of decay for the product of gene 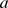. It is related to the half-life of the protein by 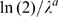. During mitosis, protein synthesis is shut down. Nuclei divide instantaneously at the end of mitosis and the protein concentrations from each mother nucleus are copied to its two daughters. We use the same division schedule as in Figure 2 of [1], which is based on [23],[42].

Gap gene circuits include cleavage cycles 13 and 14A (ending at the onset of gastrulation; 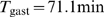) and cover the region from 35% to 92% along the A–P axis of the embryo (where 0% is the anterior pole). This includes 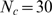 and 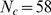 nuclei at C13 and C14A, respectively. As a consequence, system (1) consists of 120 and 232 ODEs during C13 and C14A respectively. At the boundary points 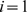 and 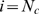 we replace the diffusion term in the right-hand side of (1) by 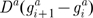 and 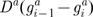 respectively, implementing homogeneous Neumann (no-flux) boundary conditions.

Kr, Kni, Gt, Tll and Hkb proteins are not present at significant levels before C13 (see Results and [10]). Thus, we use zero initial conditions for these. Non-zero initial conditions for Bcd, Cad and Hb are obtained by linear interpolation of integrated expression data at C12 (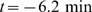) and C13 (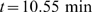). Moreover, to solve (1) one needs concentration levels 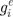 for external inputs 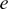 at all time points 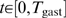. This is achieved by linear inter- or extrapolation from data points at 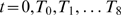 (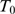 denotes the single time point in C13). Higher-order inter-/extrapolation is prone to produce artifacts due to fluctuations in the expression data, and is therefore not used here [21]. Because it is not clear whether integrated Bcd profiles at T7 and T8 have non-specific background properly removed, we used linear extrapolation based on T5/6 for these time points. This results in a rapid decay of the Bcd gradient just before the onset of gastrulation qualitatively similar to that described in [10]. Negative extrapolated concentration values were reset to zero wherever necessary.

### Parameter Estimation

Equation (1) contains 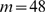 parameters (parameter vector 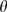 containing 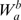, 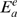, 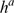, 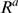, 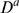 and 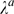), whose values we seek to determine by fitting the model to the data. We denote each measurement in our data set by 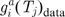, specified by the time 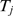 when the concentration of gene product 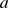 in nucleus 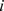 was measured. The corresponding model value obtained from (1) is denoted by 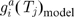. The estimation of unknown parameters in (1) amounts to minimising the cost function
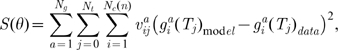
(3)where 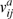 are positive weights, 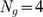 is the number of gap genes, 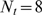 is the number of time classes, and 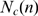 is the number of nuclei (which depends on the number of preceding mitoses 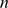) for which we have data. When all weights 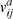 in (3) are equal to one, (3) represents an ordinary least squares (OLS) fit, which was the cost function used in all previous studies using gene circuit models [1], [7], [17]–[22]. When the weights are taken to be inversely proportional to the corresponding variances in the data, the cost function becomes the weighted least squares (WLS) distance and its minimum is the Maximum Likelihood Estimate [32].

The quality of a fit of the model to the data is measured by the root mean square (*RMS*) given by
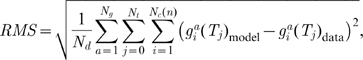
(4)where 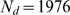 is the total number of all measurements. A solution is considered to be ‘good’ if its 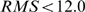 and if there are no visible pattern defects in the model response [1].

We used a two-step optimisation algorithm to minimise the cost function (3): Global optimisation by the parallel Lam Simulated Annealing (pLSA) algorithm [43]–[45] was performed on the Darwin cluster at the High-Performance Computing (HPC) centre of the University of Cambridge (http://www.hpc.cam.ac.uk) as described previously [1],[7],[21],[22]. pLSA solutions were used as starting points for local search by the Levenberg-Marquardt (LM) method [46],[47] as described in [19],[33]. The complete set of estimated parameter values can be found in Table S1. For numerical solution of the model during pLSA optimisation, we use a Runge-Kutta Cash-Karp (Rkck) adaptive-step-size solver set to high accuracy to avoid numerical instability [48]. During local optimisation by LM the model is solved using an implicit multistep Backward Differentiation Formula (BDF) as previously described in [19],[33].

Based on previous studies using gap gene circuits [1], [7], [18]–[22], we define our search space for parameter estimation by the linear constraints 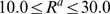, 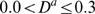, 
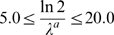
 (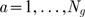), and by the following non-linear penalty function for regulatory parameters 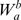 and 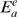


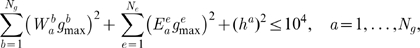
(5)where 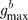 and 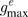 are the maximum concentration values in our data set for proteins 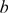 and 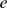, respectively. Previous work has shown that fixing the values of parameters 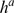 improves parameter determinability without affecting the overall quality of the fits [19]. Therefore, we take 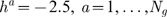 in all simulations, which leaves us with 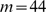 unknown parameters in (1) to be estimated.

### Statistical Analysis of Parameter Estimates

Here, we only provide a brief overview of the equations used for calculating confidence intervals and parameter correlations (see Introduction). For more detailed explanations of these statistical quantities and their derivations, we refer the reader to [19],[33] (and references therein).

Model optimisation results in a vector 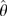 with the estimated parameter values as its elements. The ellipsoidal confidence region around 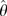, in which the ‘true’ parameter vector 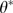 lies with a certain probability 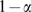 (defined as 95% in our case) is defined by
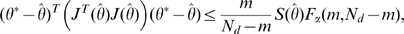
(6)where 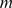 and 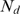 are the number of parameters and measurements, respectively. 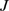 is the Jacobian (or sensitivity) matrix of size 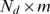, defined as 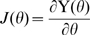 where 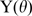 is the vector of weighted differences between model and data. Each entry 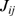 in 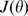 shows how sensitive the model response is at the 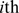 data point for a change in the 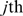 parameter. 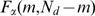 is the upper 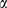 part of Fisher's distribution with 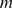 and 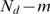 degrees of freedom. From (6) one can derive dependent and independent confidence intervals for parameter estimates 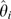 (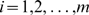). These are, respectively,
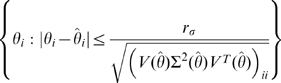
(7)and
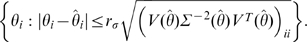
(8)Here 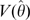 and 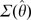 are obtained from the Singular Value Decomposition of 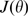 [48],[49] and 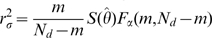.

The correlation coefficient between 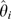 and 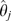 is given by
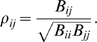
(9)where 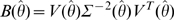.

## Results

### Quantitative Analysis of *hkb* Expression

We quantified expression levels of Hkb protein in blastoderm stage embryos of *Drosophila* as described in Methods. Our analysis closely follows that of *tll* in [10], and focuses on the last two cleavage cycles before gastrulation (C13 and C14A; cleavage cycles and time classes are defined in Methods) [23]. Representative embryo images and quantified expression patterns from those individual embryos are shown for all time classes (T1–T8) of C14A in Figure 2, left and middle column. Scaled, integrated expression data for Hkb are compared to other gap gene expression patterns in Figure 2, right column, which also indicates the number of embryos used to construct the data set.

Hkb protein can first be detected in both its anterior and posterior domain at C13 (data not shown). Protein levels rapidly increase during early C14A (T1–T3). At this stage, peak levels are very similar in both domains, although the anterior is very slightly weaker than the posterior one. Subsequently, the anterior domain gradually weakens (T5–T8), while protein levels in the posterior domain remain more or less constant (although there may be a slight decrease in concentration at T8). The peaks of both domains remain at a constant position throughout (5% A–P position for the anterior, 95% for the posterior domain). Similarly, the width of both domains remains approximately constant: the anterior domain extends back to about 10–15% A–P position, while the posterior domain reaches as far as 85–90%, both domains covering about 10–15% A–P position in each terminal region. None of the two Hkb domains show any discernible D–V asymmetry at any point in time before gastrulation.

### Model Fitting: OLS versus WLS

Our quantitative *hkb* expression data enabled us to include this gene in gap gene circuit models. We used both OLS and WLS cost functions for fitting 4-gene models (Figure 1B, right) to quantitative expression data (Figure S1). For the OLS cost function, we performed 740 independent optimisation runs (combined global and local search). The quality of a fit is assessed using the root mean square (RMS) score (defined in Methods). About 80% of the resulting parameter sets have good-scoring RMS values (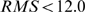). This residual error is below the level of variation in the expression data [10],[31]. However, a closer look at the patterns for good-scoring sets reveals that most of them have a slight, but significant, patterning defect in common: model output shows an artifactual hump of *Kr* expression posterior to its central domain (data not shown). This problem has also been noticed in an earlier study with gap gene circuits without *hkb* (Manu, Stony Brook University, New York, USA: personal communication). In these circuits, Gt represses *hb* and the small ectopic *Kr* domain is required to down-regulate *gt* to allow initiation of posterior *hb* expression. This is both incompatible with experimental evidence [50]–[56] and previously published models of the gap gene system [1], [7], [17]–[19],[21],[22]. Therefore, we exclude these solutions from our analysis. Although a large majority of circuits obtained by OLS fits show the small ectopic *Kr* domain, we found 39 low-scoring parameter sets that do not have this patterning defect (Figure S2). These circuits were selected for further analysis. Their 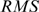 values vary between 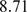 and 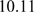.

**Figure 3:**
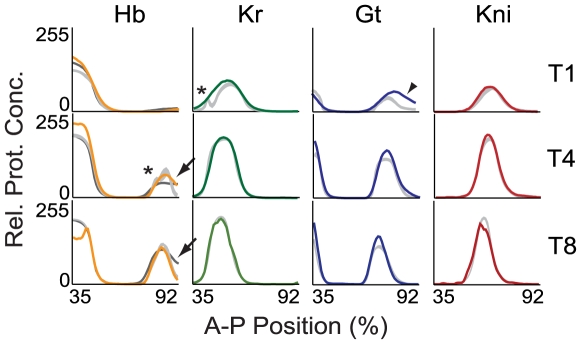
Model output compared to quantitative expression data. Integrated expression profiles from the FlyEx data base [14],[15] are shown for Hb (yellow), Kr (green), Gt (blue) and Kni (red; left to right) for time classes T2, T5 and T8 (top to bottom). Light grey profiles show corresponding profiles based on numerical solution of the current 4-gene model with parameter estimates obtained by WLS fits (see main text). The dark grey profile for Hb (left) shows model output of a representative 6-gene model from 7. Arrows highlight the correct establishment and anterior shift of the posterior boundary of the posterior *hb* domain. Patterning defects in the model are indicated as follows: Asterisks indicate bulges in the anterior borders of the central *Kr* and the posterior *hb* domain; arrowhead indicates slightly incorrect position of the early posterior border of the posterior *gt* domain. We emphasise discrepancies in boundary shape and position over those in expression levels since the latter are somewhat arbitrary due to the relative protein concentrations in the data. The incorrect reproduction of the late-appearing ‘dip’ in the anterior *hb* domain is expected, as the model currently does not include separate phases of early and late *hb* regulation (see [1] for details). Plot axes as in Figure 2, middle and right column.

Local search with the WLS cost function was performed using selected OLS parameter estimates as starting points: the 39 solutions without, and the lowest-scoring 90 solutions with defective *Kr* expression. In addition, we performed 80 independent optimisation runs using WLS both for global and local search. For our analysis, we selected 117 (out of 209) parameter sets with the lowest WLS scores varying uniformly between 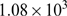 and 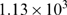. This corresponds to RMS values between 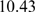 and 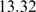, which are slightly higher than those for OLS runs since WLS solutions tolerate larger residual errors at early stages of gap gene expression. None of these low-scoring parameter sets show any major patterning defects (Figures 3 and S3), while most solutions with larger WLS scores do (data not shown). In particular, we observed no ectopic expression of *Kr* in any of these solutions. This is not surprising as standard deviations in the data are small in regions where protein concentration is low. Thus, the corresponding weights for the WLS cost function are large, which prevents the presence of any ectopic expression domains (even if they are small) in low-scoring solutions.

Gap gene expression patterns produced by circuits from the selected OLS and WLS fits are similar, although variability between different models is somewhat larger for OLS (compare Figures S2 and S3). As expected, WLS solutions generally show slightly better fits at late stages. Most visible defects occur early. The posterior borders of the central *Kr* and the posterior *gt* domain become established at a slightly different position than in the data (Figures 3, arrowhead, and S3). In addition, there are irregularities in the shape of anterior expression boundaries of the posterior *gt* domain (WLS only; Figure S3), the central domain of *Kr*, and the posterior domain of *hb* (OLS and WLS; asterisks in Figure 3). Although such irregularities in boundary shape lie well within the variability of the integrated data (cf. Figure S1), they are never observed in quantitative expression profiles extracted from individual embryos [10]. Similar problems with the posterior domains of *gt* and *hb* have been observed in earlier models of the gap gene system [1],[21].

**Figure 4:**
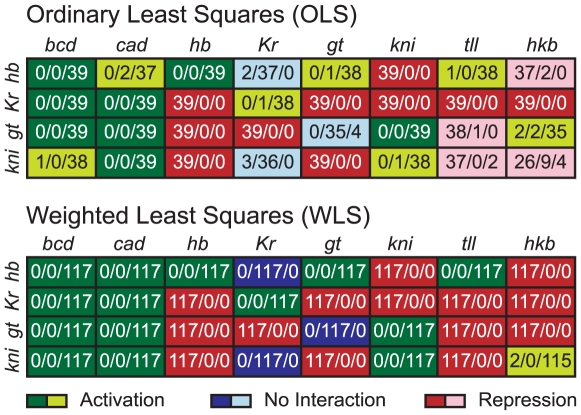
Predicted regulatory network topologies from models obtained by OLS and WLS fits. The distribution of regulatory weights for each regulator (columns) and regulated gene (rows), is shown for OLS fits (above) and WLS fits (below). Number triplets show how many parameter estimates (from independently obtained optimisation solutions) fall into the regulatory categories of ‘repression’ (parameter values 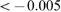; left), ‘no interaction’ (between 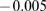 and 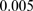; middle), and ‘activation’ (parameter values 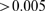; right). Background colours indicate whether a majority of the weights for a particular interaction show repression (red), activation (green) or no interaction (blue). Dark background means that all solutions fall into the same category; light colours indicate ambiguity in the prediction where some solutions fall into a different category than others. Note that the regulatory topology predicted by WLS fits with fixed Hkb weights (WLSfh) is exactly the same as that for WLS fits (not shown).

On the other hand, the dynamic expression of *hb* in its posterior domain is reproduced correctly. Earlier models exhibited defects in the timing and positioning of the posterior boundary of this domain (see dark grey Hb profile in Figure 3), while the circuits presented here accurately reproduce the establishment and subsequent anterior shift of this expression border (arrows in Figure 3).

### Consistency of Parameter Estimates

Estimates of regulatory weights obtained by both OLS and WLS fits were classified into the following three categories: ‘activation’ (parameter values 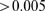), ‘repression’ (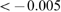) and ‘no interaction’ (between 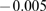 and 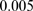) [1],[18],[19]. This leads to a predicted regulatory topology of the network based on which category a majority of parameter estimates falls into (summarised in Figure 4). If a threshold of 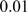 is chosen instead, the predicted network topology remains largely unchanged, with two notable exceptions: the activating effects of both Cad and Tll on *hb* change to the ‘no interaction’ category indicating that these predicted interactions are very weak, and may not be significant (see Discussion).

Apart from only two interactions, the predicted regulatory topologies agree between OLS and WLS fits. In the case of OLS, Hkb activates *gt* and represses *kni*, while for WLS it is the other way around (Figure 4). Strikingly, the more consistent expression patterns between WLS solutions are also reflected by more consistent predictions of network structure. While many parameters fall into different categories in different OLS solutions, only one interaction (regulation of *kni* by Hkb) shows this type of ambiguity in the case of WLS (Figure 4). This means that WLS solutions are not only more tightly clustered in terms of their expression patterns, but also in terms of the distribution of their parameter values.

A similar pattern can be observed when comparing our new 4-gene models with earlier 6-gene circuits (cf. Figure 1B). Although the predicted regulatory structure is largely in agreement between these two types of model, consistency of the prediction is improved considerably in 4-gene models (even in the case of the OLS solutions presented here). Repression of *Kr* and *gt* by Hb, of *kni* by Gt, of *Kr* by Kni and of *gt* by Tll are now present in all parameter sets, while previous results for the 6-gene case showed no interaction for these weights in many solutions [1],[18],[19]. Weak activation of *hb* by Tll is now predicted by a large majority of parameter sets. Some previous models had predicted this interaction [17], while most showed repression or no interaction between the two genes [1],[18],[19]. Another activating interaction which is now consistently predicted is that between Kni and *gt*. Finally, there is no auto-activation of *gt* in a very large majority of our parameter sets.

### Parameter Determinability

The regulatory structure of the gap gene system shown in Figure 4 is based solely on the classification of estimated parameters into regulatory categories. To assess the quality of the parameter estimates more rigorously, we computed dependent and independent confidence intervals for each parameter set (see Methods and [19],[33]). We then checked if these confidence intervals fall entirely into negative (‘repression’), or positive (‘activation’) ranges of parameter values, or whether they cluster tightly around zero (‘no interaction’).

**Figure 5:**
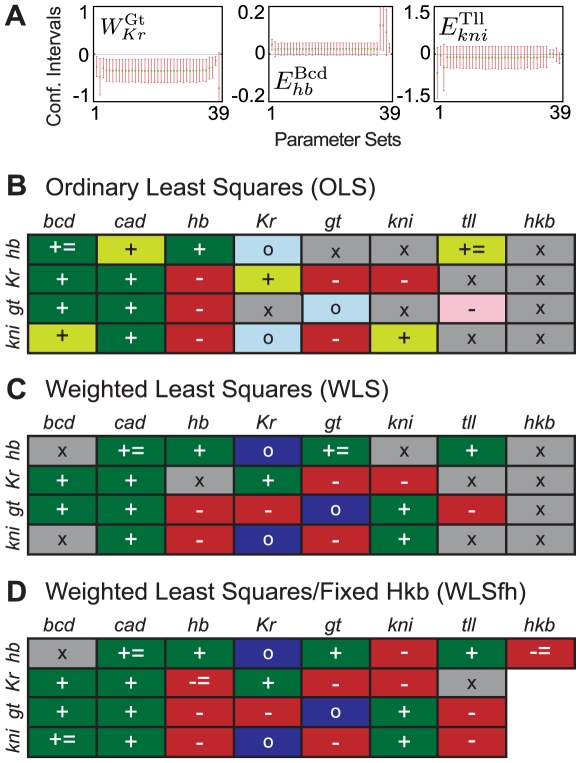
Parameter determinability analysis. (A) Dependent (green) and independent (red) confidence intervals are shown across 39 OLS solutions (horizontal axes) to illustrate a regulatory weight which is well determined (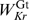, left), one that is only weakly determined (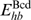, middle), and one that is not determined at all (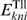, right). Vertical axes represent parameter values; note that scales vary between plots. (B–D) Summaries of parameter determinability analysis for OLS (B), WLS (C) and WLS fits with fixed Hkb weights (WLSfh; D). Symbols indicate whether a particular interaction between a regulator (columns) and a regulated gene (rows) is well determined (-, repression; +, activation; o, no interaction), only weakly determined (- = , no activation; + = , no repression), or not determined at all (x). Background colours as in Figure 4B–D, except that grey indicates non-determinability. See text for equations and details.

Results in Figure 4 are fully confirmed when only dependent confidence intervals (which tend to underestimate the extent of the confidence region) are taken into account. In contrast, not all of our conclusions from Figure 4 are supported when independent confidence intervals (which tend to overestimate the extent of the confidence region) are considered. For example, Figure 5A shows the confidence intervals for interactions between Gt and *Kr* (left; parameter: 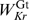), Bcd and *hb* (middle; 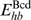), as well as Tll and *kni* (right; 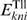) for all 39 selected OLS fits. Independent confidence intervals for 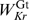 lie in the negative part of the plane for almost all parameter estimates and therefore, repression predicted for this weight in Figure 4 is confirmed by statistical analysis. In other words, this parameter is determinable. Independent confidence intervals for 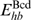, on the other hand, slightly extend into the negative part of the plane. Therefore, the model only predicts that Bcd does not repress *hb*. Note that this is a weaker conclusion than predicting activation for this weight from Figure 4. Hence, this parameter is only weakly determinable. In contrast, we cannot draw any conclusions about 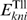, since independent confidence intervals extend from the negative into the positive part of the plane. Thus, statistical analysis cannot confirm the repression of *kni* by Tll inferred from Figure 4, and this parameter is not determinable.

Parameter determinability analysis based on independent confidence intervals for OLS and WLS fits is summarised in Figures 5B and 5C, respectively. We focus on regulatory parameters since, just as in earlier studies [19], promoter strengths 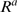, diffusion coefficients 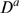 and decay rates 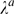 have extremely large independent confidence intervals meaning that none of these parameters are determinable (data not shown). Confidence intervals for all regulatory weights are shown in Figures S4 (for OLS) and S5 (for WLS fits). It is evident that conclusions from this analysis are generally weaker than those drawn from classifying parameter values only (compare Figures 5B,C with Figure 4).

11 and 12 (out of 32) regulatory parameters cannot be determined for OLS and WLS fits, respectively. Among them are several of the interactions predicted to fall into the ‘no interaction’ category in Figure 4 (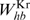, 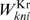 and 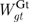) if a threshold of 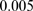 is chosen for the analysis. However, independent confidence intervals of these interactions are all very small and cluster tightly around zero (Figures S4 and S5). Furthermore, their intervals are completely within the ‘no interaction’ category if the threshold is extended to 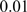. For these reasons, we consider them to be determinable in Figure 5B and C. This lowers the number of non-determinable regulatory parameters to 10 for both OLS and WLS fits. Out of the remaining 22 regulatory weights, 2 are only weakly determinable (for both OLS and WLS fits), while the regulatory category for the other 20 is confirmed by statistical analysis. Which regulatory parameters are not determinable differs significantly between OLS and WLS solutions and does not follow any obvious pattern, apart from the fact that most interactions by terminal gap genes *tll* and *hkb* are affected (Figure 5B,C).

### Regulation of the Posterior *hb* Domain

Previous quantitative analyses of the gap gene system suggested a set of basic regulatory mechanisms based on broad activation of gap genes by maternal co-ordinate proteins, and spatially specific gap-gap cross-repression [1],[7]. In addition, they revealed significant anterior shifts in the position of posterior gap domains after their initial establishment during C13 [7],[10]. These shifts are caused by asymmetric repressive interactions as described in the Introduction and in [1],[7],[22]. Parameter analysis (Figures 4 and 5), as well as graphical inspection of regulatory interactions across space and time (data not shown; analysis performed as in [1],[7]) show that our current 4-gene models implement exactly the same regulatory principles as those seen in previous 6-gene circuits.

**Figure 6:**
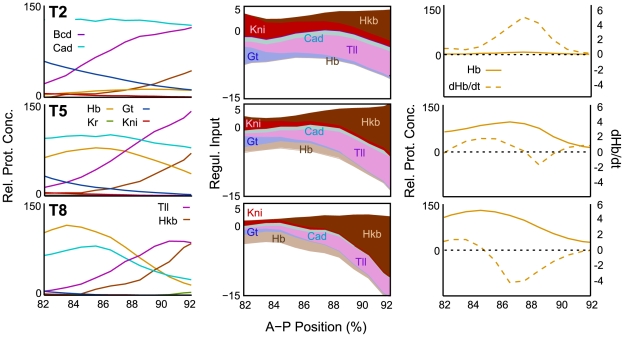
Graphical regulatory analysis of the posterior *hb* domain. Expression profiles from the model (left), regulatory contributions (middle) and change in Hb protein concentration (dashed) vs. Hb protein levels (solid lines; right) are shown in the posterior region of the embryo. Horizontal plot axes represent percent A–P position as in Figure 2. Vertical axes represent relative protein concentrations (left and right columns), regulatory contributions (coloured areas are given by 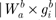 or 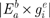 in equation (1) and reflect the strength of a given interaction at a specific point in space and time; contributions by repressors are shown in dark, activators in light colour; middle column), or relative change in protein concentration over time (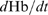; right column). All plots are based on our best scoring WLS solution (circuit WLS57; see Table S1 for parameter values). Other solutions showed equivalent mechanisms (data not shown). Here, we focus on *hb* activation and the regulation of the posterior boundary of this domain. For an analysis of the anterior boundary, see [7] (Supplementary Information, Figure 14).

In addition, our current gap gene circuits now accurately reproduce expression in the posterior *hb* domain, while shift and establishment of this domain were incorrect in previous models [1], [7], [17]–[22] (Figure 3). To investigate how the inclusion of Hkb affects this domain, we have performed a detailed graphical analysis of *hb* regulation in the posterior region of the embryo (Figure 6). This analysis reveals the following regulatory principles.

The posterior *hb* domain is the last gap domain to form in the posterior region of the embryo. Expression is initiated during cleavage cycle 13 and the domain retracts from the posterior pole in early cycle 14A (T2) [10],[57],[58]. Later during cycle 14A, expression levels increase, domain boundaries sharpen and shift further towards the anterior (see Figures 2 and 6, left column).

The late initiation of *hb* expression in the posterior region can be explained by residual amounts of Kni protein being present in the region during C13 and early cycle C14A (Figure 6, T2, left and middle panel). Kni is a very strong repressor of *hb*. Kni is increasingly repressed in the most posterior region of the embryo by the gradual accumulation of Gt protein (data not shown). In the model, combined activating inputs by Cad and Tll induce *hb* expression where Kni levels have fallen to a low-enough level (Figure 6, T2, middle and right panel). At later stages, *hb* auto-activation gradually supplements and replaces activation by other factors (Figure 6, T5/T8, middle). The posterior boundary of the posterior *hb* domain is set by Hkb repression (Figure 6, T2–T8, middle). The accumulation of Hkb in this region causes an increase in both levels and extent of this repression over time. This in turn leads to an anterior shift in the region where *hb* is expressed, such that Hb protein is only actively produced in the anterior part of its domain, while protein degradation dominates further posterior (Figure 6, T5–T8, right). At this level, the mechanism underlying the shift in the posterior *hb* domain is equivalent to those of other gap domains [7]: expression can extend anteriorly due to the lack of repression by the adjacent domain (posterior *gt*), while it becomes increasingly repressed posteriorly (by Hkb, in this case).

### Models with Fixed Hkb Regulatory Parameters

Our analysis of parameter determinability indicates that those parameters with particularly large confidence intervals could be fixed to specific values—within the non-empty intersections of their dependent intervals—without affecting the quality of the fits. Diffusion rates, for example, show large confidence intervals, despite not being significantly correlated with other parameters (see also below). Therefore, fixing their values during optimisation (to averaged values based on previously found estimates: 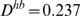, 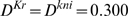, and 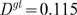) will not change the determinability of the remaining parameters but will reduce the size of the optimisation problem. On the other hand, regulatory weights describing the effect of Hkb on *Kr*, *gt* and *kni* have large confidence intervals (see Figures S4 and S5) because of correlations to other parameters, in particular the regulatory effects of Tll on the same targets (data not shown). This indicates a certain level of redundancy. Since a large majority of the dependent confidence intervals for these weights cover negative *and* positive values, we have set all of them to zero during optimisation. This leaves us with 37 parameters to be re-estimated.

We used local search with 60 initial parameter sets arbitrarily chosen from the previously found 117 WLS parameter sets. Additionally, we performed 20 global optimisation runs with these parameters fixed. From the resulting solutions, we selected 66 circuits which have low WLS values (about 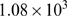). As expected, expression patterns produced by these models are very similar to those for WLS fits (data not shown).

The network topology shown for WLS runs in Figure 4 remains absolutely unchanged for the new estimates (with the obvious exception of the regulatory parameters for regulation of *Kr*, *gt*, and *kni* by Hkb which have been set to zero; data not shown). We calculated confidence intervals for these solutions to test whether more parameters are determinable in these models than in OLS and WLS fits with Hkb weights included (Figure S6). Our analysis, based on independent confidence intervals, is summarised in Figure 4D. It is immediately evident that determinability of regulatory parameters has significantly improved in these circuits compared to WLS fits. Only 2 weights (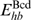 and 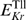) remain non-determinable, 4 show weak determinability (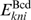, 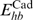, 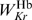 and 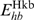), while for the other 23 the confidence intervals confirm the type of regulation revealed by parameter classification. This is a significant improvement compared to circuits which include all regulatory weights for Hkb (compare Figure 4B,C with 4D).

### Parameter Correlations

The occurrence of non-determinable parameters is often caused by correlations between parameters [19],[34]. We have analysed these correlations for WLS models with fixed Hkb regulatory parameters, by calculating the mean correlation matrix for all parameters across solutions (see Methods and Figure S7). Note that for all significant entries of the mean correlation matrix the standard deviation is negligible, meaning that those correlations are present in all individual correlation matrices. This revealed the following correlations for parameters which are not or only weakly determinable in these models: Activation of *hb* by Bcd is negatively correlated with the activating effects of Cad (also weakly determined) and Gt, which indicates a certain level of redundancy of these interactions in the model. Repression of *Kr* by Tll is negatively correlated with activation of *gt* by Cad, indicating that the more Gt there is in the posterior (through increased activation of *gt* by Cad), the less repression by Tll is required to keep *Kr* expression off in the posterior of the embryo. The repression of *hb* by Hkb is negatively correlated with activation of *hb* by Tll, which indicates that a balance needs to be maintained between these interactions to enable correct posterior *hb* expression.

Finally, the last two interactions which are only weakly determined are the activation of *kni* by Bcd (negatively correlated with repression of *kni* by Hb) and the repression of *Kr* by Hb (negatively correlated with activation of *Kr* by Bcd; Figures 4D and S7). A similar correlation between Bcd activation and Hb repression can also be seen for *gt*, but does not lead to reduced determinability in this case. Similar correlations were also found in earlier 6-gene models [19]. They corroborate results which indicate that a delicate balance between activation and repression is essential for correct gap gene expression in the trunk region of the embryo [2]. In addition, we find similar negative correlations between Tll repression and Cad activation for the posterior gap genes *gt* and *kni* (Figure S7). These do not affect parameter determinability in our current models, but did so in earlier 6-gene models [19]. This indicates that balance between activation and repression through different maternal systems is crucial in the posterior region of the embryo as well.

### Prediction of Mutant Expression Patterns

After regulatory weights of gap gene circuits have been estimated based on wild-type expression data, analysis of mutants can be conducted *in silico* [59]. Null mutants of any regulator 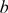 (or 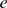) can be simulated by setting regulatory weights 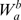 (or 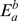) to zero for all regulated genes 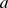 (while leaving all other parameter values unchanged). Similar to earlier gap gene circuit models [1], our current models do not reproduce expression patterns in mutant backgrounds for *hb*, *Kr*, *gt* or *kni* correctly (data not shown). In contrast, we were more successful at simulating null mutants of the terminal gap genes *tll* and *hkb*.

**Figure 7:**
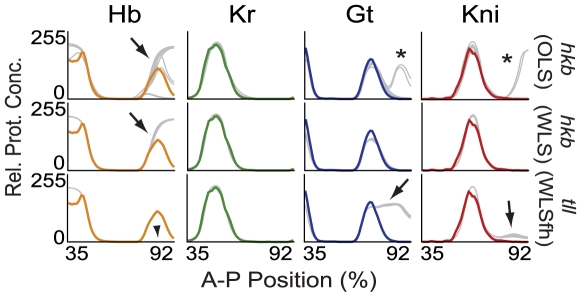
Simulation of terminal gap gene mutants. Simulated expression profiles of Hb, Kr, Gt and Kni (left to right) in *hkb* (top: OLS, middle: WLS) and *tll* mutant backgrounds (bottom: WLS with fixed Hkb weights) are shown at time class T8. Plot axes and colours as in Figure 3: wild-type data shown in colour, mutant model output in grey. Arrows indicate de-repression of posterior gap gene expression, arrowhead absence of the posterior *hb* domain; both consistent with experimental evidence. Asterisks indicate posterior de-repression of *gt* and *kni* in OLS circuits inconsistent with published experimental observations.

The only known alteration of gap gene expression in *hkb* mutants is the failure of posterior *hb* to retract from the posterior pole [26],[60]. This is reproduced correctly in both OLS and WLS solutions (arrows in Figure 7, upper and middle row). In addition, however, many OLS solutions show de-repression of *gt* and *kni* in posterior regions of the embryo (asterisks in Figure 7), which is inconsistent with the evidence. We never observed such defects in WLS circuits.

Embryos mutant for *tll* show more severe patterning defects: Both the posterior domain of *gt* and the abdominal domain of *kni* are expanded posteriorly [26],[53],[54],[61],[62], while the posterior *hb* domain is reduced or absent in these embryos [26],[60],[63]. Only *Kr* does not seem to be affected [63],[64]. Most OLS and WLS solutions show mutant expression patterns which are inconsistent with this evidence (data not shown). Surprisingly, however, circuits obtained by WLS with fixed diffusion rates and Hkb regulatory parameters, reproduce these defects correctly: there is no posterior *hb* expression (arrowhead in Figure 7), while *gt* and, to a lesser degree, *kni* are de-repressed in the posterior region of the embryo (arrows in Figure 7, bottom row).

## Discussion

Our results constitute a comprehensive, integrative analysis of the expression and function of the terminal gap gene *hkb* in the blastoderm embryo of *Drosophila*. On one hand, we have characterised the expression of *hkb* in a quantitative manner. On the other, we used a systems-level approach—the gene circuit method—to show how Hkb exerts its effect on the expression of *hb* in its wild-type genetic context, and to demonstrate that it does not have any non-redundant function in gap gene regulation beyond that. But before we discuss these biological insights in more detail below, we highlight two significant improvements in the gene circuit methodology, which have important implications for reverse engineering biological networks in general.

### Methodological Improvements

First, we were able to increase the efficiency of optimisation, and the consistency of parameter estimates, by using weighted least squares (WLS) instead of ordinary least squares (OLS) for optimisation. The use of a WLS cost function also reduces the need for human intervention when selecting solutions for analysis, since it prevents the occurrence of minor (but biologically significant) patterning defects such as the ectopic *Kr* domain observed in most OLS solutions. Out of 740 optimisation attempts with OLS, we only obtained 39 biologically realistic models. In contrast, none of the WLS solutions exhibited this problem, and thus a much larger proportion of them were suitable for analysis. This constitutes a very drastic increase in overall computational efficiency and biological relevance of the obtained fits. Furthermore, OLS solutions showed much larger variability in expression patterns and parameter values than those obtained with WLS. This indicates that fitting with WLS to data with non-constant standard deviations not only leads to biologically more relevant, but also to more consistent results across optimisation runs.

Second, analysis of parameter determinability [19],[34] allows us to eliminate parameters from the optimisation problem, thereby considerably reducing the complexity of the problem. Our models have 48 parameters, a number which we managed to reduce to 37 by fixing non-determinable parameters to specific values (see also [19]). Statistical analysis based on confidence intervals not only gives us an indication of which parameters to fix, but also of which values to fix them to (see Results). This was used successfully here for both diffusion rates and regulatory parameters representing the regulatory effect of Hkb on its targets *Kr*, *gt* and *kni*. Not only were we able to reduce the computational effort for optimisation, but fixing parameters also significantly improved parameter determinability, such that only 2 out of 29 regulatory parameters now remain non-determinable. This is a vast improvement over previous, 6-gene models [19].

### Expression and Regulation of *hkb*

In terms of the biology, we first discuss the expression and regulation of *hkb*. Our quantitative analysis of *hkb* expression confirms and extends results from earlier studies. Both Hkb domains cover about 10–15% A–P position in the anterior and posterior terminal region of the embryo [26],[39],[40]. Their borders coincide with the limits of the invaginating mesoderm in the ventral furrow during gastrulation [39]. Expression of *hkb* is more restricted to the terminal regions of the embryo than expression of *tll* (see Figure 2, right column). This difference is very clear at all time points for the posterior domains. In contrast, the early anterior domains of Hkb and Tll are very similar in extent, and only diverge at mid C14A (from about T5 onward), when the anterior Tll domain retracts from the pole. There are other, more subtle differences as well, revealed by a comparison with the quantitative analysis of Tll in [10]: The anterior domain of Hkb appears before that of Tll, which can only be detected during early cycle 14A. Hkb levels in this domain also decrease much earlier again (from T5 onward) than those of Tll in its anterior domain, whose peak levels remain constant until right before the onset of gastrulation (T7/8). Finally, the anterior domain of Hkb does not show any D–V asymmetry before gastrulation, while the corresponding domain of Tll retracts from the anterior pole and becomes increasingly dorsal during late cycle 14A (T5–T8). In contrast, dynamics of the maximum protein level in the posterior Hkb domain closely follows that of Tll, with the only potential difference being that Hkb persists very slightly longer in this region than Tll right before the onset of gastrulation (T8).

These results are entirely consistent with what we know about *hkb* regulation. The expression of *hkb* is completely independent of any other gap genes (including *tll*) [26],[28]. Both *hkb* domains depend on higher levels of Torso signalling from the terminal maternal system than those of *tll*, explaining their more restricted spatial extent [39], [65]–[67]. In addition, the anterior domain also requires the presence of Bcd [68]. These activating inputs are enabled through local relief of strong repression mediated by ubiquitous maternal factors such as Dead ringer (Dri) and Groucho (Gro) in the terminal regions of the embryo [69]. Interestingly, *hkb* is also regulated by the D–V maternal system, which is required for the ventral shift of the anterior *hkb* domain during gastrulation [28],[69]. Our results clearly indicate that this interaction is not significant before gastrulation as we can detect no D–V asymmetry in any of the two *hkb* domains at this stage (Figure 2).

### Regulation of Trunk Gap Genes

**Figure 8:**
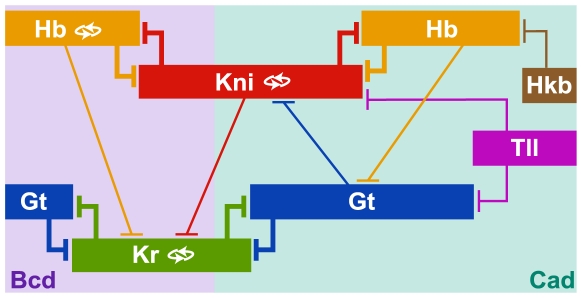
Summary of predicted gap gene regulatory mechanisms. Gap domains are shown schematically, with anterior to the left, posterior to the right. Background colours indicate the most prominent activating input to each domain. Auto-activation is indicated by double-arrows. T-bars indicate repressive gap-gap cross-regulation (thickness of the bars indicates repressive strength). See text for details.

But how does Hkb affect regulation of other gap genes? The regulatory mechanisms for the expression of the trunk gap genes *hb*, *Kr*, *gt* and *kni* predicted by our models are summarised in Figure 8: (1) Gap genes are broadly activated by the maternal gradients of Bcd and Cad. (2) Auto-activation is involved in maintenance and sharpening of boundaries in the anterior domain of *hb*, the central domain of *Kr* and the abdominal domain of *kni*. (3) The basic staggered arrangement of gap domains is provided by mutual repression between non-overlapping gap genes *hb* and *kni*, as well as *gt* and *Kr*. (4) Asymmetric repression between overlapping gap genes leads to anterior shifts in domain positions. (5) Terminal gap genes *tll* and *hkb* repress gap gene expression in the posterior terminal region of the embryo. These regulatory principles largely confirm results from previous studies using gap gene circuits [1], [7], [17]–[19],[21],[22].

The most significant improvement of our models over earlier ones is that they now correctly reproduce the expression and shift of the posterior *hb* domain (Figure 3). This means that our current models now reproduce the dynamic shifts of all posterior gap domains correctly [10]. Our analysis suggests that the appearance of this domain depends on the retraction of Kni—through increasing repression by Gt—from the posterior terminal region of the embryo in early cycle 14A (Figure 6). Its posterior boundary is set and subsequently shifted by increasing Hkb repression. These regulatory mechanisms are strongly supported by experimental evidence. Kni has been shown to repress *hb*: mis-expression of *kni* leads to a reduction of *hb* expression in the affected regions [70]–[72], and the posterior *hb* domain expands anteriorly in *kni* mutants [72]. Moreover, the abdominal domain of *kni* is expanded posteriorly in *gt* mutants [53], and reduced in embryos over-expressing *gt* [73]. Finally, repression of *hb* by Hkb is supported by the fact that the posterior *hb* domain fails to retract from the posterior pole in *hkb* mutant embryos [26],[60].

While our models reproduce repressive effects on posterior *hb* expression in a way consistent with experimental evidence, there is not much convincing data supporting the activating inputs responsible for posterior *hb* expression predicted by our models. Accordingly, we have omitted them from our regulatory summary in Figure 8. Both activation of *hb* by Cad and by Tll are predicted to be weak in the model. In the case of Cad, there is no evidence for any interaction with *hb*, as *hb* is expressed normally in mutants lacking both zygotic and maternal *cad* [29], as well as in embryos over-expressing *cad* [74]. Activation by Tll seems to be supported by the fact that posterior expression of *hb* is strongly reduced or absent in *tll* mutant embryos [26],[60],[63],[75], while the posterior *hb* domain expands anteriorly in embryos over-expressing *tll* [75]–[77]. In addition, there are several predicted Tll binding sites in the regulatory element responsible for posterior *hb* expression [78]. On the other hand, there is strong evidence that activation of *hb* by Tll is largely indirect—via repression of *kni* by Tll—as posterior *hb* expression is present in *tll kni* double mutants [77]. Finally, there is some evidence against a role of *hb* auto-activation in the posterior region. Mutants that express a non-functional Hb protein show no obvious defects in posterior *hb* expression [79]. Moreover, the expression of *hb* reporter constructs in the posterior *hb* domain is broadened and more intense in a *hb* mutant background compared to wild-type, while it is strongly reduced in embryos over-expressing *hb* [75]. In the model, none of these activating contributions provide any spatial specificity to posterior *hb* expression, which is mainly due to repression by Kni and Hkb (Figure 6). Taken together, this suggests that *hb* may be activated by an unknown, uniformly expressed maternal factor in this region.

There is another unresolved question concerning the posterior *hb* domain: Translation of *hb* is repressed by the posterior gradient of the maternal co-ordinate protein Nanos (Nos) and its co-factors [80]–[82]. These factors act through a Nos-response element, which is present in both maternal and zygotic transcripts of *hb* [81]. It remains unclear how this translational repression is overcome during mid cycle 14A. Either, the Nos gradient has disappeared (or is disappearing) by this time (this has never been assessed), or enough *hb* transcripts must accumulate to overcome Nos' repressive effect on translation. Quantitative studies of the Nos gradient will be required to resolve this issue.

For the posterior domain of *hkb*, our results show conclusively that its effect on *hb* expression is the only role it plays in gap gene regulation in the wild-type embryo. Excluding interactions of Hkb with *Kr*, *kni* and *gt* has no effect on any of these genes in the model. In fact, parameter determinability and prediction of *tll* mutant gene expression patterns improve significantly if these interactions are excluded (Figures 5 and 7). However, there is some evidence suggesting that Hkb does repress *Kr* and *gt*: The central *Kr* domain expands further posterior in embryos mutant for the maternal gene *vasa (vas)*, *tll* and *hkb* than in those mutant for *vas* and *tll* alone [83]. Similarly, the posterior domain of *gt* expands further posterior in *tll hkb* double mutants than in embryos mutant for *tll* alone [26],[28]. Furthermore, the posterior *gt* domain is absent in embryos over-expressing *hkb* [28],[39]. Note that all of this evidence comes from over-expression experiments or embryos mutant for multiple genes, including *tll*. This suggests that there are two main reasons why interactions of Hkb with *Kr*, *gt* and *kni* do not play a role in the wild-type embryo: First, expression of *hkb* never overlaps its potential target genes (with the exception of *gt*; Figure 2). And second, its repressive input seems to be completely redundant with the corresponding repressive contributions by Tll. This is confirmed by our analysis of parameter determinability.

Apart from the regulation of the posterior *hb* domain, there are only two predicted interactions that differ in our 4-gene models compared to those in earlier 6-gene models. First, there is no auto-activation of *gt* in a large majority of our parameter sets. Although this interaction was present in earlier models [1], [17]–[19], gap gene auto-activation in general is not required for correct gap gene expression [17]. Second, activation of *gt* by Kni is supported by the fact that the posterior domain of *gt* is weakened and its posterior border fails to form properly in *kni* mutant embryos [52]–[54]. Fits in which this interaction is fixed to zero all show the ectopic expression of *Kr* described for OLS fits in the Results section, indicating that it is necessary for correct regulation of *gt* in the model (data not shown). However, it remains unclear whether its inclusion is an improvement over previous models. The experimental evidence remains ambiguous (effects are weak and the affected posterior border of *gt* occurs in a region where *kni* is not expressed), and activation of *gt* by Kni causes the transient patterning defect observed in the anterior border of the posterior *gt* domain in our current models (Figure S3). In summary, this suggests that neither of these two differences significantly affect the biological relevance of the models.

Little is known about the function and effect of the anterior *hkb* domain. In particular, it is not known why anterior *hkb* does not seem to have a repressive effect on *hb*, as both genes are co-expressed in this region. Unfortunately, we have not been able to include this domain in our analysis since our models currently do not include head gap genes, which are essential for patterning in the anterior region of the embryo.

### Simulating Mutants

Apart from correct posterior *hb* expression, the second major improvement of the models reported here is that they are able to reproduce null mutants of the terminal gap genes *tll* and *hkb* (see Figure 7). A theoretical study previously established that, in principle, it is possible to predict mutant patterns based on gene circuit fits to wild-type data only [59]. However, earlier gene circuit models—optimised against real, noisy expression data—failed to correctly reproduce any gap gene null mutants so far (including *tll* mutants) [1]. Our models provide an important first step towards the solution of this problem.

Apart from mutations in *hkb* and *tll*, gap gene circuit models have been shown to correctly reproduce gap gene expression (and its variational properties) in the presence of fluctuations in the Bcd gradient [21],[22]. All of these perturbations affect the gap gene network in a feed-forward manner. Neither *bcd*, *hkb* nor *tll* are regulated by gap genes themselves.

On the other hand, our current models still cannot accurately reproduce null mutants of the trunk gap genes *hb*, *Kr*, *gt* and *kni* (data not shown). All of these genes regulate and are regulated by other gap genes. This indicates that the problem is connected with feedback regulation within the model. Various potential reasons for this have been proposed in the past: over-simplified representation of transcriptional regulation in the model, missing production delays, scaling problems in the data, over-fitting to noisy expression data, or missing factors in the model which are redundant in the wild-type, but become important in a mutant background [1]. Further systematic studies will be necessary to elucidate which of these factors affect feedback regulation in our models in a way which makes them fail to reproduce such mutant expression patterns.

### Developmental and Evolutionary Implications

Why is all this important? After all, our results establish that *hkb* plays a very minor role in gap gene regulation. Yet, understanding the regulatory function of *hkb* is crucial for a better understanding of both the developmental and evolutionary dynamics of the gap gene system. Our current models are the first to reproduce all shifts of posterior gap domains correctly. There is evidence suggesting that the mechanism underlying these shifts is an emergent property of the entire gap gene network [7],[22]. If this is correct, we cannot understand gap domain shifts completely without understanding how *all* of these domains are regulated.

This view is supported by the following: First, there are no known mutants that affect any of the gap domain shifts individually. Moreover, evidence from an analysis of the dynamical behaviour of gap gene circuits suggests that all trunk gap genes participate in the shift mechanism in an integrated way [7],[22]. Repression between overlapping gap domains (as described in the Introduction) interacts in complex ways with the mutually repressive interactions between *Kr* and *gt* as well as *hb* and *kni*. In addition, terminal gap genes contribute to domain shifts as well, as we have established in this study. We are far from understanding the causal flow of regulatory information in this system. Our analysis suggests that the posterior *hb* domain may play a central role in it. All posterior nuclei in the system converge towards an attractor state in which *hb* is expressed at high level [22]. Moreover, the delayed establishment of its posterior domain coincides precisely with the phase of development when domain shifts occur. Further evidence from *tll* mutant embryos, which lack a posterior *hb* domain, will be required to better understand the causal role of this domain in gap gene regulation.

Changes in the regulation of the posterior *hb* domain also play an important role in the evolution of the gap gene system in dipteran insects (flies, midges, and mosquitoes). Primitive, nematoceran flies such as the psychodid midge *Clogmia albipunctata* lack posterior *hb* expression before gastrulation [84], while the posterior domains of *gt* and *hb* appear to have swapped positions in mosquitoes [85]. It will be interesting to investigate whether gap gene regulation in these embryos requires Hkb, and how the absence (or change in position) of posterior gap domains affects boundary shifts and their regulation compared to *Drosophila*. It has been noted previously that shifting gap domains are reminiscent of travelling waves of gene expression in animals with sequential segment determination [1], which is widely assumed to be the ancestral state of segment determination (reviewed in [86]). This suggests that shifting domains are ancestral as well. Understanding how regulatory changes in posterior *hb* expression affect these shifts in various dipteran species will not only help us understand how the gap gene network performs its patterning function, but also how it evolved. In view of this, our models are an important first step towards an integrative, systems-level understanding of the developmental and evolutionary dynamics of the gap gene network.

## Supporting Information

Figure S1 Gap gene expression data used for model fitting. Integrated expression patterns (dark lines) with corresponding standard deviations (lightly coloured areas) are shown for Hb (red), Kr (green), Kni (purple) and Gt (blue) at cleavage cycle 13 (C13) and eight time classes (T1–8) during cleavage cycle 14A. Relative protein concentrations are plotted against percent A–P position (where 0% is the anterior pole). All patterns shown are from the FlyEx data base: http://urchin.spbcas.ru/flyex. See Methods for details on data processing.
(0.41 MB PDF)

Figure S2 Model output compared to quantitative expression data (OLS fits). Integrated expression profiles from the FlyEx data base (http://urchin.spbcas.ru/flyex) are shown for Hb (yellow), Kr (green), Gt (blue) and Kni (red; left to right) for time classes C13 and T1–T8 (top to bottom). Grey profiles show corresponding profiles based on numerical solution of the model with parameter estimates obtained by OLS fits. Relative protein concentrations are plotted against percent A–P position (where 0% is the anterior pole).
(0.51 MB PDF)

Figure S3 Model output compared to quantitative expression data (WLS fits). Integrated expression profiles from the FlyEx data base (http://urchin.spbcas.ru/flyex) are shown for Hb (yellow), Kr (green), Gt (blue) and Kni (red; left to right) for time classes C13 and T1–T8 (top to bottom). Grey profiles show corresponding profiles based on numerical solution of the model with parameter estimates obtained by WLS fits. Relative protein concentrations are plotted against percent A–P position (where 0% is the anterior pole).
(0.95 MB PDF)

Figure S4 Parameter determinability analysis: confidence intervals for OLS fits. Columns represent regulators, rows regulated genes. Dependent (green) and independent (red) confidence intervals are shown across all selected 39 OLS solutions (horizontal axes). Vertical axes represent parameter values; note that scales vary between plots.
(0.64 MB PDF)

Figure S5 Parameter determinability analysis: confidence intervals for WLS fits. Columns represent regulators, rows regulated genes. Dependent (green) and independent (red) confidence intervals are shown across all selected 117 WLS solutions (horizontal axes). Vertical axes represent parameter values; note that scales vary between plots.
(1.03 MB PDF)

Figure S6 Parameter determinability analysis: confidence intervals for WLS fits with fixed Hkb weights (WLSfh). Columns represent regulators, rows regulated genes. Dependent (green) and independent (red) confidence intervals are shown across all selected 66 WLSfh solutions (horizontal axes). Vertical axes represent parameter values; note that scales vary between plots.
(0.73 MB PDF)

Figure S7 Mean correlation matrix for WLS fits with fixed Hkb weights (WLSfh). Parameter correlations are arranged in blocks per regulated gene. Abbreviations indicate regulator (for regulatory weights) or parameter (for promoter strength and decay rates). Positive correlations are shown in green, negative correlations in blue. For clarity, only correlation values above 0.5 are shown. Note that most correlations occur between parameters involved in the regulation of the same gene (diagonal blocks of the matrix).
(0.42 MB PDF)

Table S1 Estimated parameter values are shown for all OLS, WLS and WLSfh optimisation runs.
(0.11 MB ODS)
