# Supplementary figures and images for: Gene Circuit Analysis of the Terminal Gap Gene huckebein
Source: PLoS Comput Biol. 2009 Oct 30;5(10):e1000548. doi: 10.1371/journal.pcbi.1000548 (PMC2760955; doi:10.1371/journal.pcbi.1000548)

Expression Data (with Standard Deviations) used for Model Fitting

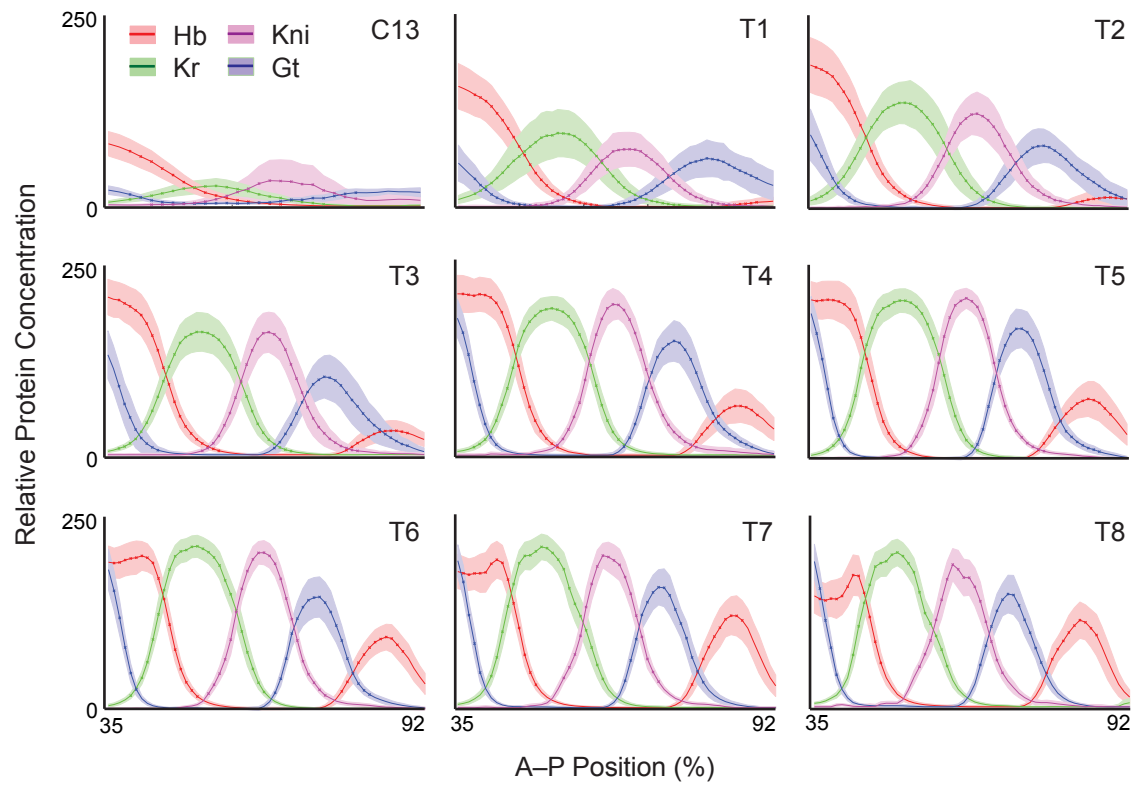

**Figure S1**

Supplement: Figure S1 — Gap gene expression data used for model fitting. Integrated expression patterns (dark lines) with corresponding standard deviations (lightly coloured areas) are shown for Hb (red), Kr (green), Kni (purple) and Gt (blue) at cleavage cycle 13 (C13) and eight time classes (T1–8) during cleavage cycle 14A. Relative protein concentrations are plotted against percent A–P position (where 0% is the anterior pole). All patterns shown are from the FlyEx data base: http://urchin.spbcas.ru/flyex. See Methods for details on data processing. (0.41 MB PDF) [file pcbi.1000548.s001.pdf]

Ordinary Least Squares (OLS) Solutions (N=39)

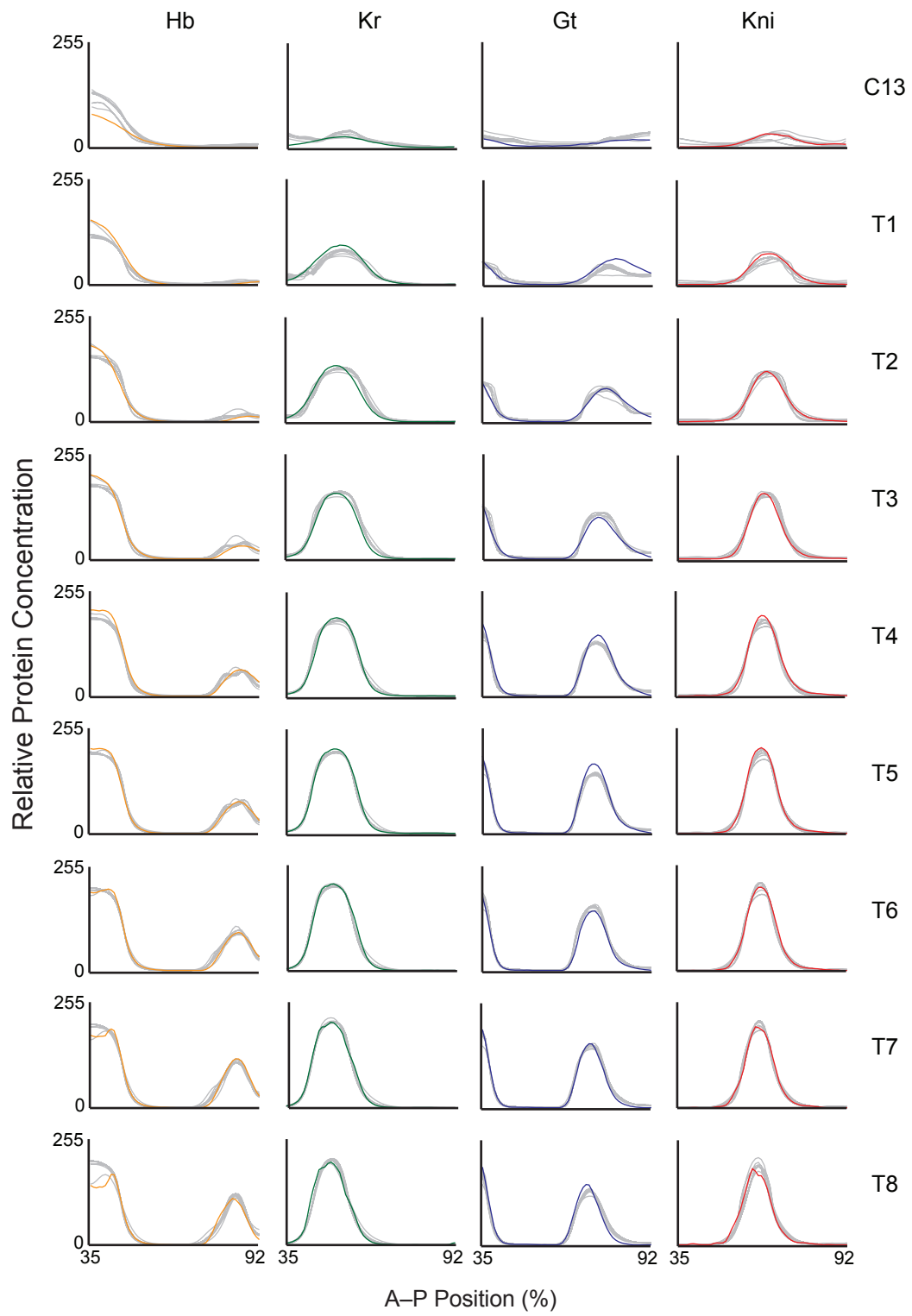

**Figure S2**

Supplement: Figure S2 — Model output compared to quantitative expression data (OLS fits). Integrated expression profiles from the FlyEx data base (http://urchin.spbcas.ru/flyex) are shown for Hb (yellow), Kr (green), Gt (blue) and Kni (red; left to right) for time classes C13 and T1–T8 (top to bottom). Grey profiles show corresponding profiles based on numerical solution of the model with parameter estimates obtained by OLS fits. Relative protein concentrations are plotted against percent A–P position (where 0% is the anterior pole). (0.51 MB PDF) [file pcbi.1000548.s002.pdf]

Weighted Least Squares (WLS) Solutions (N=117)

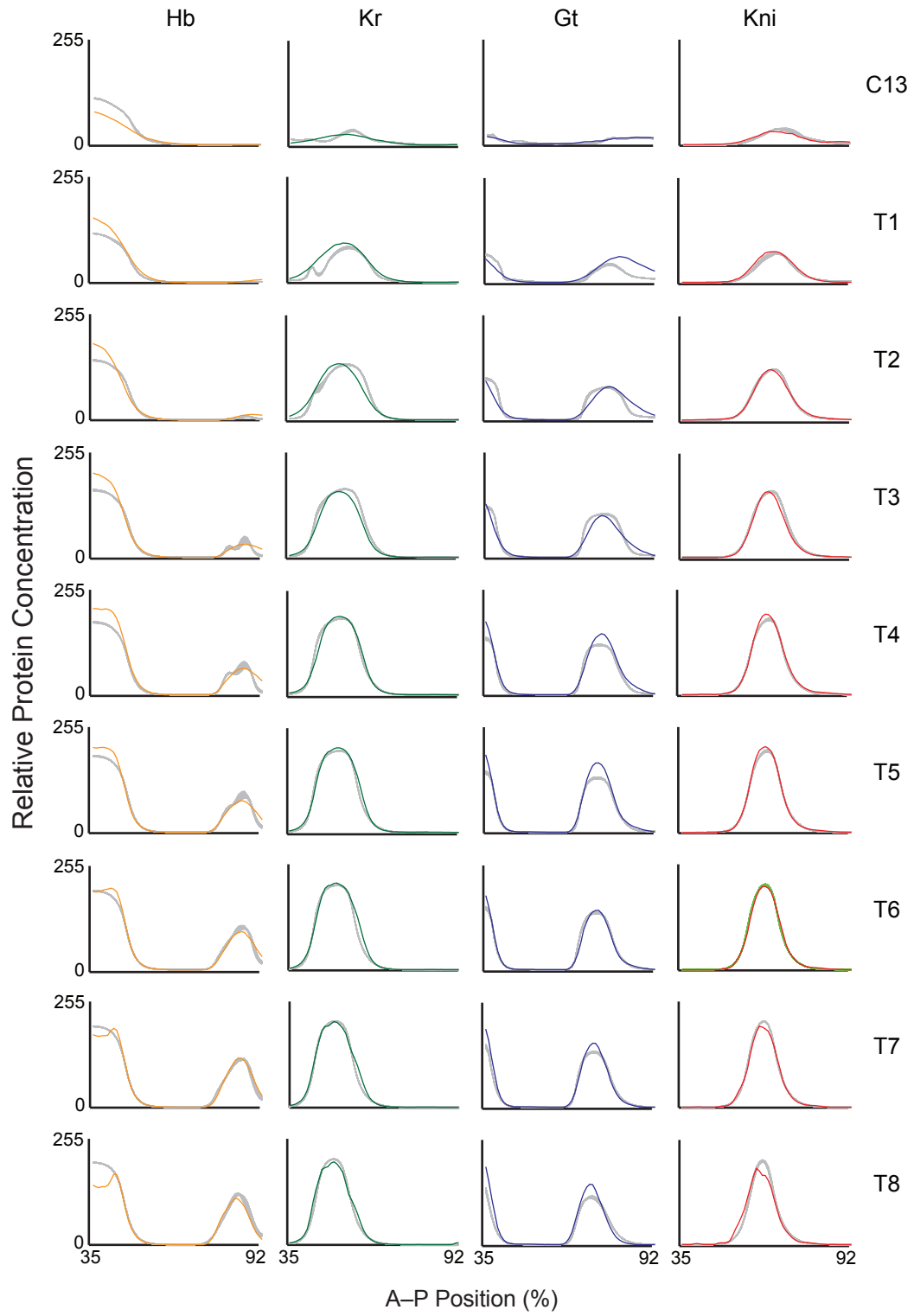

**Figure S3**

Supplement: Figure S3 — Model output compared to quantitative expression data (WLS fits). Integrated expression profiles from the FlyEx data base (http://urchin.spbcas.ru/flyex) are shown for Hb (yellow), Kr (green), Gt (blue) and Kni (red; left to right) for time classes C13 and T1–T8 (top to bottom). Grey profiles show corresponding profiles based on numerical solution of the model with parameter estimates obtained by WLS fits. Relative protein concentrations are plotted against percent A–P position (where 0% is the anterior pole). (0.95 MB PDF) [file pcbi.1000548.s003.pdf]

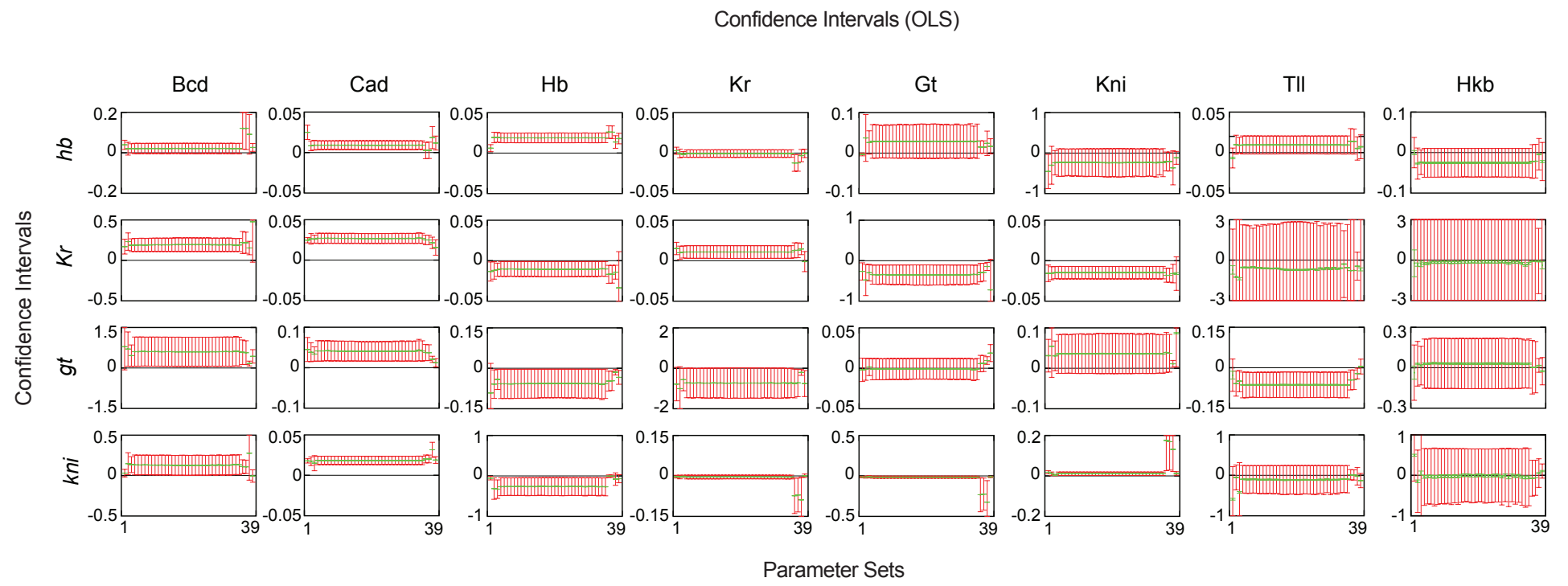

**Figure S4**

Supplement: Figure S4 — Parameter determinability analysis: confidence intervals for OLS fits. Columns represent regulators, rows regulated genes. Dependent (green) and independent (red) confidence intervals are shown across all selected 39 OLS solutions (horizontal axes). Vertical axes represent parameter values; note that scales vary between plots. (0.64 MB PDF) [file pcbi.1000548.s004.pdf]

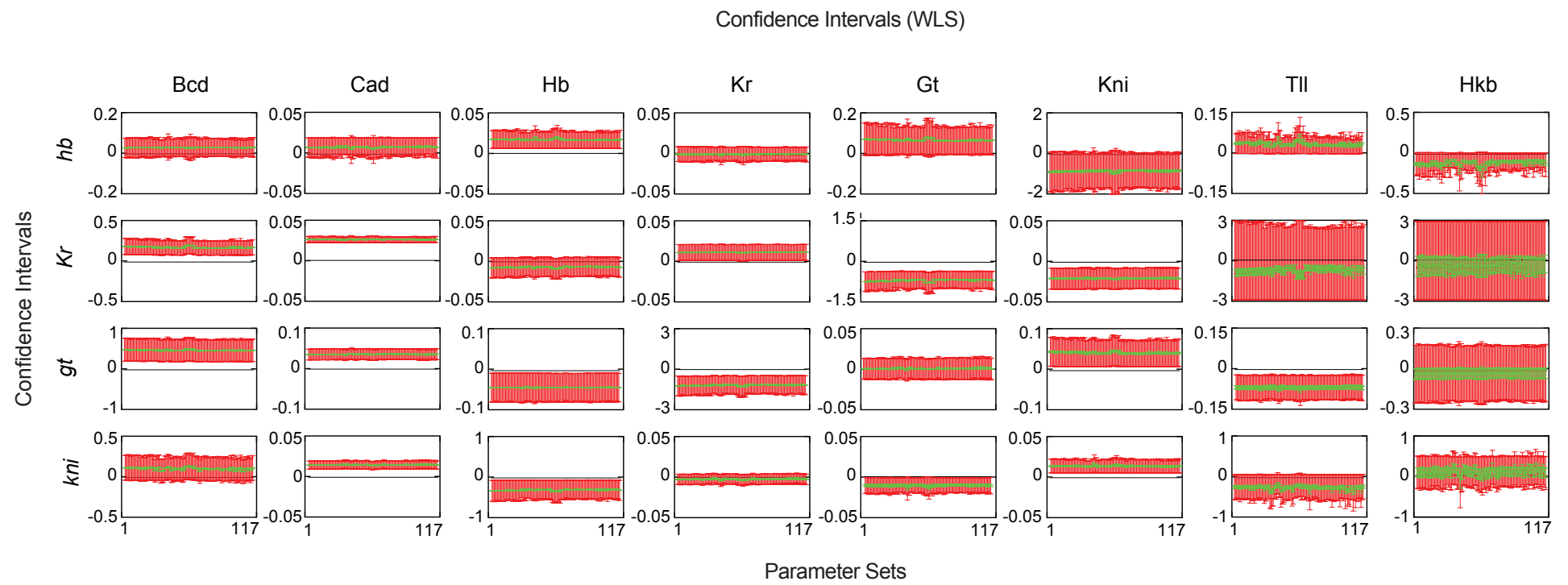

**Figure S5**

Supplement: Figure S5 — Parameter determinability analysis: confidence intervals for WLS fits. Columns represent regulators, rows regulated genes. Dependent (green) and independent (red) confidence intervals are shown across all selected 117 WLS solutions (horizontal axes). Vertical axes represent parameter values; note that scales vary between plots. (1.03 MB PDF) [file pcbi.1000548.s005.pdf]

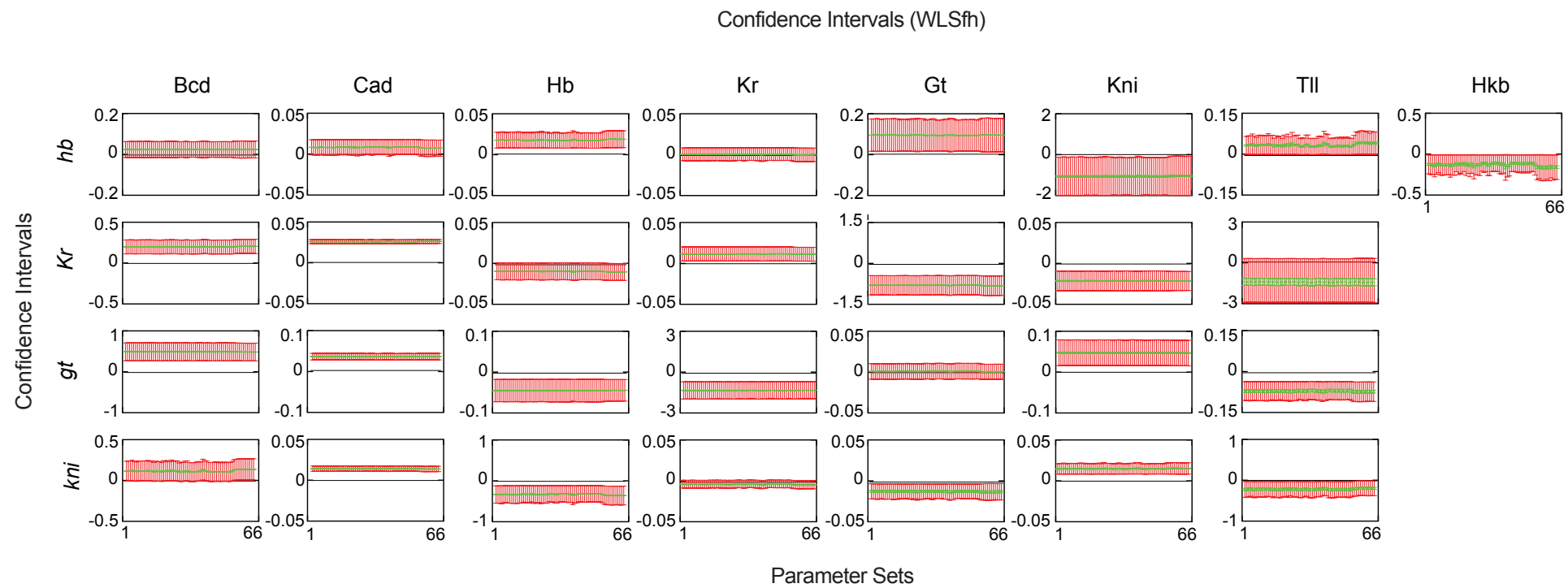

**Figure S6**

Supplement: Figure S6 — Parameter determinability analysis: confidence intervals for WLS fits with fixed Hkb weights (WLSfh). Columns represent regulators, rows regulated genes. Dependent (green) and independent (red) confidence intervals are shown across all selected 66 WLSfh solutions (horizontal axes). Vertical axes represent parameter values; note that scales vary between plots. (0.73 MB PDF) [file pcbi.1000548.s006.pdf]

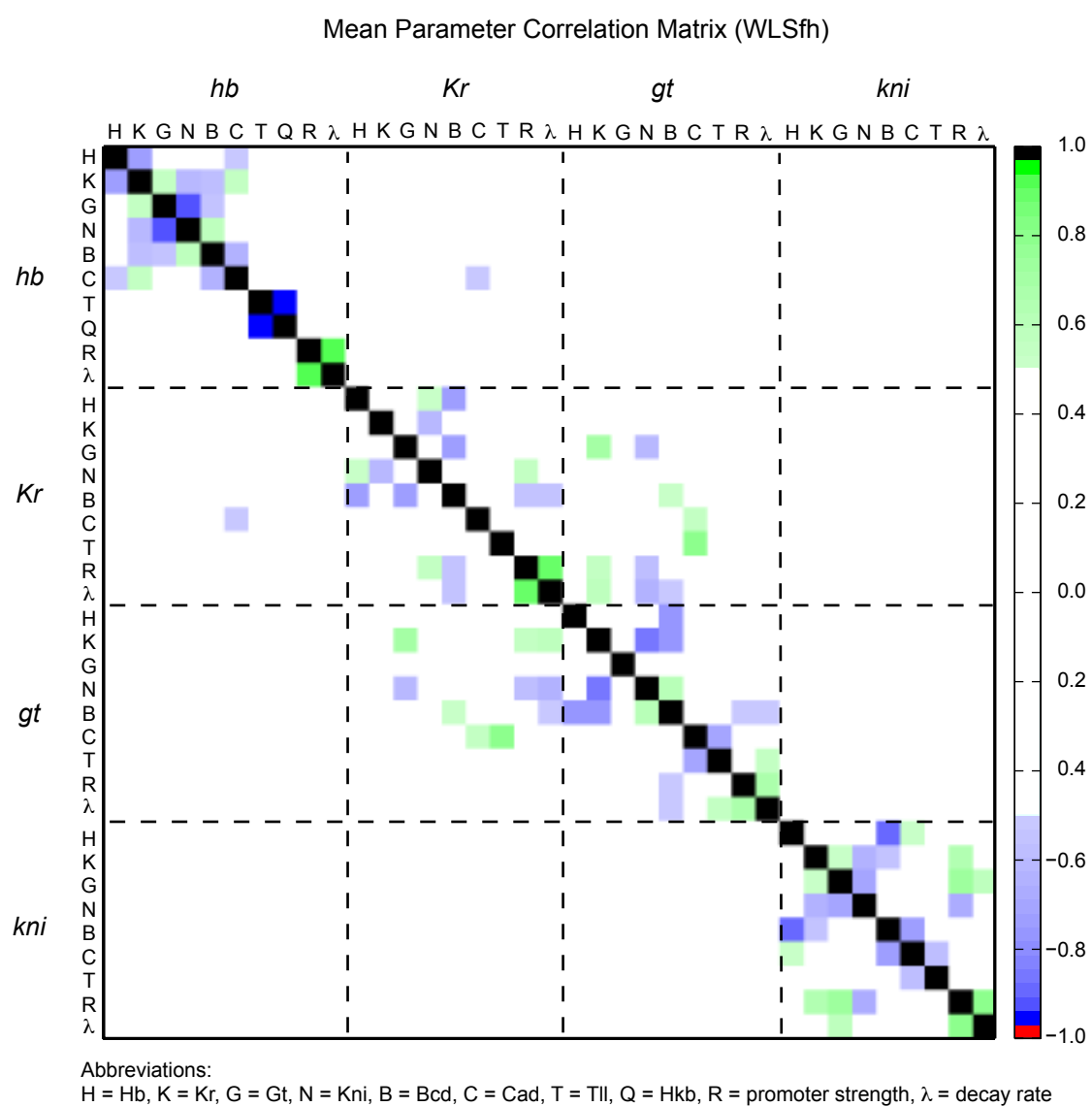

**Figure S7**

Supplement: Figure S7 — Mean correlation matrix for WLS fits with fixed Hkb weights (WLSfh). Parameter correlations are arranged in blocks per regulated gene. Abbreviations indicate regulator (for regulatory weights) or parameter (for promoter strength and decay rates). Positive correlations are shown in green, negative correlations in blue. For clarity, only correlation values above 0.5 are shown. Note that most correlations occur between parameters involved in the regulation of the same gene (diagonal blocks of the matrix). (0.42 MB PDF) [file pcbi.1000548.s007.pdf]
